# Drug resistance in cancer therapy: the Pandora's Box of cancer stem cells

**DOI:** 10.1186/s13287-022-02856-6

**Published:** 2022-05-03

**Authors:** Hamed Rezayatmand, Mahboobeh Razmkhah, Iman Razeghian-Jahromi

**Affiliations:** 1 School of Pharmacy, Kyiv Medical University, Kyiv, Ukraine; 2 Shiraz Institute for Cancer Research, Shiraz University of Medical Sciences, Shiraz, Iran; 3 Cardiovascular Research Center, Shiraz University of Medical Sciences, 3rd Floor, Mohammad Rasoolallah Research Tower, Namazi Hospital, Shiraz, Iran

**Keywords:** Treatment resistance, Drug resistance, Chemotherapy resistance, Cancer stem cell, Mechanism

## Abstract

Drug resistance is the main culprit of failure in cancer therapy that may lead to cancer relapse. This resistance mostly originates from rare, but impactful presence of cancer stem cells (CSCs). Ability to self-renewal and differentiation into heterogeneous cancer cells, and harboring morphologically and phenotypically distinct cells are prominent features of CSCs. Also, CSCs substantially contribute to metastatic dissemination. They possess several mechanisms that help them to survive even after exposure to chemotherapy drugs. Although chemotherapy is able to destroy the bulk of tumor cells, CSCs are left almost intact, and make tumor entity resistant to treatment. Eradication of a tumor mass needs complete removal of tumor cells as well as CSCs. Therefore, it is important to elucidate key features underlying drug resistance raised by CSCs in order to apply effective treatment strategies. However, the challenging point that threatens safety and specificity of chemotherapy is the common characteristics between CSCs and normal peers such as signaling pathways and markers. In the present study, we tried to present a comprehensive appraisal on CSCs, mechanisms of their drug resistance, and recent therapeutic methods targeting this type of noxious cells.

## Introduction

Cancer treatment has reached promising breakthroughs during the last decades [1]. Despite all these progresses, chemoresistance has been remained as the main hurdle to achieve success in eliminating cancer cells [2]. Chemoresistance, which means non-optimal respond to chemical drugs, limits drug efficacy [3]. Indeed, chemoresistance is associated with transformation of tumor cells into a more aggressive and/or metastatic forms, [4, 5] and it is considered as the main reason of death in cancer patients [6]. About nine out of ten cancer deaths are due to spreading cancer cells from the primary tumor mass to local and remote tissues (metastasis) [7].

Stem cells (SCs) maintain tissue homeostasis using the unique property of self-renewal [8]. Drug resistance and cancer relapse are occurred due to the presence of a type of stem cells called cancer stem cells (CSCs) within a tumor with the ability to generate heterogeneous lineages of cancer cells based on their self-renewal and differentiation potential [9]. Following chemotherapy, the density of CSCs within the tumor is enriched because CSCs are able to survive and proliferate even after eradication of the majority of cancer cells [10]. Recent findings revealed that chemotherapy induces reprogramming or differentiation of normal cancer cells toward generation of CSC-like cells [11]. Another challenge in cancer therapy is the presence of heterogenous cells in the tumor. These cells have different morphology and proliferative index, are genetically variable, and dissimilar in responding to chemotherapy agents [12]. Multidrug resistance, which means resistance to a broad spectrum of agents, is frequently seen for tumor eradication at the clinical level [13]. Drug resistance could be represented either intrinsically, inherent resistance to drugs, or acquired, which emerges after exposure of tumor cells to chemicals [14]. CSCs have mechanisms that show endogenous resistance at a much higher degree than normal tumor cells [15]. There is an intense need to have a clear picture of the features and mechanisms of resistance employed by CSCs [9].

## CSCs

In the nineteenth century, Conheim declared that dormant embryonic stem cells become active, and start to proliferate after certain stimulations leading to the formation of large masses of tumors [16]. This is for the first time that such a tumor-developing role has been assigned to stem cells. Later in 1994, Lapidot and coworkers isolated CSCs from peripheral blood of patients with leukemia. Implantation of the isolated cells into mice generates human leukemia [17]. In the following years, identification of CSCs in breast tumors and other solid tumors such as brain cancer, colorectal cancer, and liver cancer were reported [18]. High number of CSCs increases proliferation capacity, the risk of poor clinical outcome, and genetic instability.

Although CSCs are a small population of cells in the cancerous entity [19], CSCs clusters harbor a huge potential of metastasis, 25 to 50 folds higher than CSCs alone [20]. They justify their appellation due to their similar characteristics with normal stem cells including ability to self-renew, differentiation, and expression of surface stemness markers [21].

The origin of CSCs is not well-understood as they possess a dynamic state. Interestingly, a non-CSCs population could produce CSCs, and a population with high density of CSCs could generate non-CSCs [22]. CSC plasticity within a tumor is largely influenced by some factors such as context and environment [23]. These cells may originate from normal stem cells that become tumorigenic because of genetic or environmental changes. An alternative theory is that the differentiated cells transform into cancer cells with stem-like properties [24]. Others believe that epigenetic plasticity that is often represented in epithelial-mesenchymal-transition (EMT) is involved in the generation of CSCs. In EMT, epithelial characteristics like cell–cell adhesion is lost, and mesenchymal traits like increased motility and invasiveness are gained [25]. Cancer cells show stem cell-like properties such as invasion to neighboring tissues and resistance to therapeutics upon EMT [26]. These alterations are mainly determined epigenetically through methylation of DNA, modifications of histone, and differential genes expression. Notably, EMT is not a biphasic process, and dynamic transitional states such as hybrid epithelial/mesenchymal state could be deployed by the cells [27]. Lack of precision about the origin of CSCs makes many researchers to use alternative terms like cancer stem-like cells or tumor initiating cells [2].

## Mechanisms of drug resistance by CSCs

**Fig. 1:**
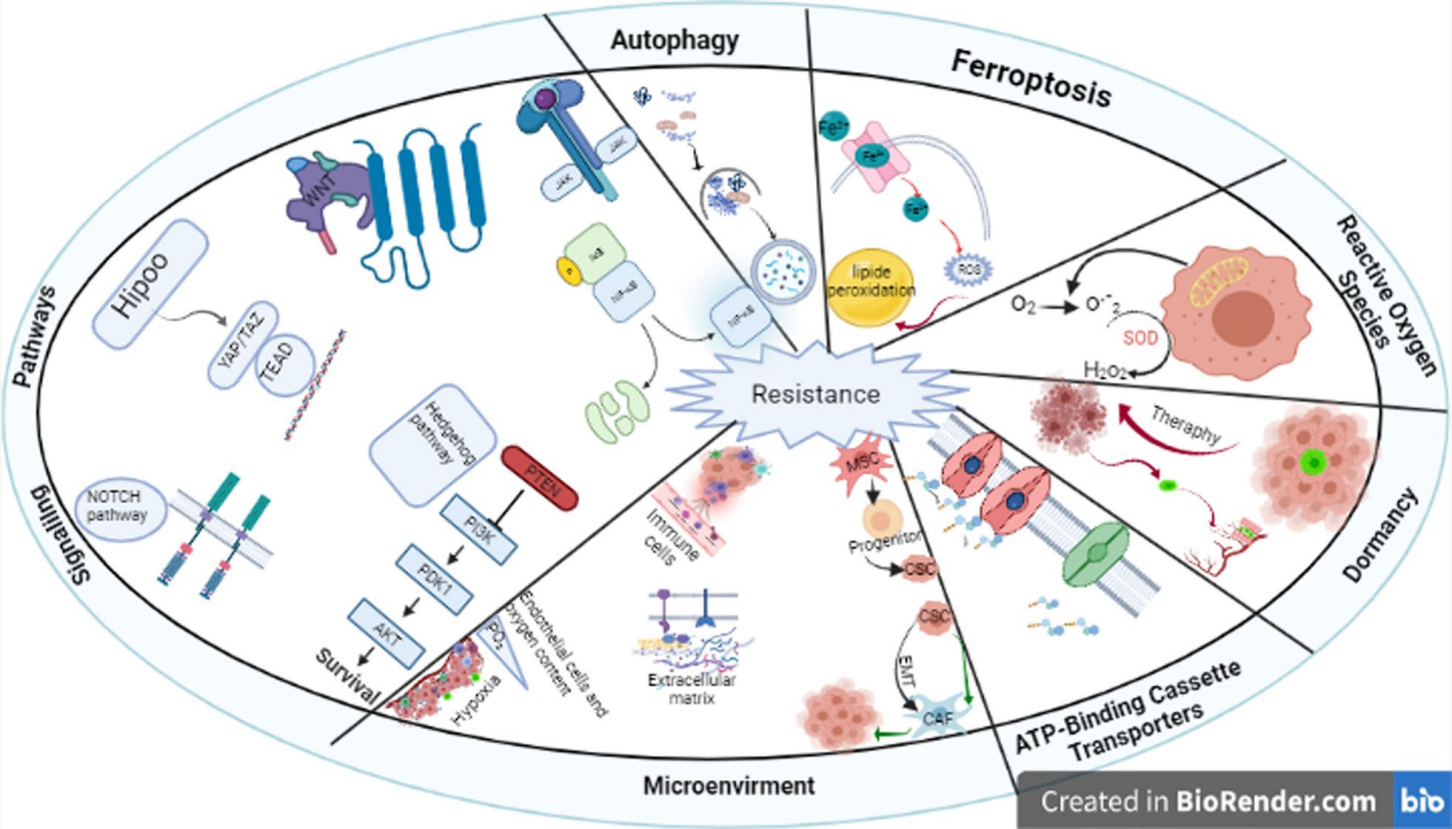
Different mechanisms used by CSCs to resist against chemotherapy

Different intrinsic and extrinsic factors regulate CSCs functions. Intrinsic regulators are genetic, epigenetic, and metabolic effectors while extrinsic regulators are microenvironment and the immune system [28]. Signaling pathways like those in normal stem cells are also found in CSCs though deregulated in some cases [29]. Discovery of CSCs has revolutionized the understanding of how tumors are formed, and it shed light into new avenues of therapy and prognosis. The presence of CSCs now explains the heterogeneous nature of many tumors [30]. Having efficient diagnostic and therapeutic methods in the battle against cancer needs in-depth perception of the mechanisms of resistance used by CSCs. CSCs use several ways to resist against chemical agents in cancer therapy (Fig. 1).

### Signaling pathways

CSCs and other stem cells like that of embryonic ones have common characteristics particularly developmental signaling pathways. These highly conserved pathways controls self-renewal of the stem cells [31]. Activation of such pathways expand CSCs yielding resistance to therapy [32]. Wnt/β-catenin, Hedgehog, Notch, Janus kinase-signal transducer and activator of transcription (JAK-STAT), Nuclear factor erythroid 2-related factor 2 (NRF2), and Hippo-YAP/TAZ play critical roles in CSCs [2]. However, they are not tightly regulated in these cells. This dysregulation is one of the underlying reasons of distinction in proliferation, metastasis, and resistance to treatment between cancer and normal stem cells [33]. For instance, deregulated Notch signaling stimulates self-renewal in CSCs in breast cancer and oral squamous cell carcinoma. Furthermore, interconnection of signaling pathways with each other affects the function of the downstream effectors S. For instance, Notch pathway is influenced by signal transfer between Wnt and Hedgehog pathways [34]. Other signaling pathways like Transforming growth factor beta (TGFβ), phosphatidylinositol 3-kinase/protein kinase B (PI3K/Akt), and epidermal growth factor receptor (EGFR) along with transcriptional regulators such as SRY-Box Transcription Factor 2 (SOX2), cellular Myc (c-Myc), NANOG, and Octamer-binding transcription factor 4 (OCT4) are active in CSCs to maintain self-renewal and differentiation properties [35]. It was shown that Wnt, Notch, Hedgehog, and Yes-associated protein 1/ Transcriptional coactivator with PDZ-binding motif (YAP/TAZ) signaling pathways contribute substantially to metastasis [36].

#### Wnt pathway

Wnt pathway regulates cell proliferation, survival, and cell fate as well as embryonic development. Asymmetrical cell division, cell polarity, and cell migration are all under the control of Wnt pathway. This pathway supports somatic stem cells in a variety of tissues [12], and in combination with Notch pathway regulates the expression of the main markers of stemness like c-Myc [37]. Wnt pathway is abnormally activated in many cancers [38]. For instance, nearly 90% of patients with colon malignancy show dysregulated high Wnt signaling in their CSCs [39]. Overactivation of Wnt/B-catenin is a hint for discrimination of tumor cells and differentiated cells [40].

Resistance to combination therapy of IFN-α/5-FU results from activation of Wnt/β-catenin signaling [41]. Endogenous activation of Wnt/β-catenin signaling in OV6^+^ cells makes them resistant to standard chemotherapy [42]. Nuclear β-catenin translocation and transactivation of Wnt genes like multidrug resistance gene 1, an important player in chemoresistance, activates Wnt/β-catenin pathway in neuroblastoma cancer cells due to overexpression of frizzled-1 Wnt receptor resulting in attenuation of sensitivity to chemotherapy [43]. In ovarian cancer, c-Kit, which is a stem cell factor receptor, activates Wnt/β-catenin and ATP-binding cassette G2 pathway producing chemoresistance [44]. CD44^+^/CD133^+^ CSCs are considerably associated with high expression of Wnt pathway in patients with colorectal cancer [45]. Wnt signaling was associated with CSC-related metastasis in breast cancer. Proteins of Wnt signaling like LEF1, cyclin D1, β-catenin, and TCF-4 are highly expressed in breast CSCs in comparison with non-stem cancer cells. In this regard, CSC population as well as stemness-mediated genes including CD44, ALDH1, and Sca-1 were downregulated upon Wnt1 knockdown [46] (43 as 2).

#### Notch pathway

Proliferation, differentiation, apoptosis, and intercellular communication of normal stem cells are controlled by Notch signaling pathway. This pathway also regulates survival and self-renewal of CSCs. Upregulated Notch components were seen in CSCs of pancreas tumor [12]. Notch-1 pathway activates nuclear factor kappa light chain enhancer of activated B cells (NF-kB) followed by stimulation of CSC to proliferate via downregulation of some anti-apoptotic proteins like survivin and B-cell lymphoma 2 [47]. Activation of Notch-1 signaling leads to EMT phenotype in those lung cancer cells that are resistant to gefitinib [48, 49]. In glioma, CD133^+^ CSCs contribute to the activation of genes involved in Notch and Hedgehog pathways, and gives resistance against temozolomide treatment [50]. Chemical drugs such as oxaliplatin increases γ-secretase activity and instigates Notch-1 receptor and its upcoming target Hes-1 in colon cancer. Hence, inhibitors of γ-secretase is a good alternative to sensitize colon cancer cells to chemotherapy [51]. Tumorsphere formation by CSCs was increased or decreased by activation or knock down of Notch target gene (Hes1), respectively [52]. Maintenance of CSCs and their resistance to platinum are regulated by Notch signaling pathway, particularly Notch3, in ovarian cancer [53]. Cisplatin therapy activates Notch signaling, and enriches CD133^+^ cells in lung adenocarcinoma [54].

According to a study on 115 samples of breast tumor tissues, Notch activity were significantly associated with ALDH1 expression [55]. Notch signaling was associated with overactivation of iota, a protein kinase C, that plays role in survival of stem-like cells in glioblastoma. Deactivation of iota causes proliferation reduction and apoptosis of glioblastoma CSCs [56].

#### Hedgehog pathway

This pathway controls several cellular and molecular processes during embryogenesis as well as development and homeostasis of adult tissues. Hedgehog pathway also regulates CSCs in different cancer types [12]. In fact, self-renewal property is maintained in CSCs relying on overexpression of the Hedgehog pathway [8]. It was shown that Hedgehog inhibitors decrease proliferation, survival, self-renewal, and clonogenicity of CSCs in glioma. Furthermore, expression of stemness genes like NANOG, SOX2, and OCT4 are decreased upon inhibition of Hedgehog pathway [57]. Regarding Hedgehog signaling, the expression of SMO (a G protein–coupled receptor protein) is inversely related to the CSCs activity in chronic myeloid leukemia in a way that SMO knockout leads to CSCs enhancement and disease progression [58]. Hedgehog ligand in breast cancer cells reprograms cancer fibroblasts to activate FGF5 expression and fibrillary collagen production in order to support niche giving resistance to therapy [59].

#### PTEN/PI3K/Akt pathway

This pathway controls many important physiological and pathological processes like cell proliferation, angiogenesis, metabolism, differentiation, and survival. Mutations of phosphatase and tensin homolog (PTEN) are evident in more than half of the glioblastoma multiforme specimens [12]. Interestingly, stemness genes (OCT4, SOX2, and NANOG) are expressed in PTEN-knock out neural stem cells. These cells retain differentiation capability, and produce different cell lineages. They also acquire neoplastic phenotype including increased growth, resistant to death, and elevated migration potential along with in vivo invasiveness [12].

One of the functions of PI3K/Akt pathway is regulation of ABCG2 activity through its homing in the plasma membrane. The side population phenotype of glioma cancer stem-like cells is promoted thereafter due to PTEN loss [60]. With regard to the role of Notch pathway in resistance of glioma stem cells, γ-secretase inhibitors cause glioma stem cells to be more vulnerable to therapy because of PI3K/Akt activation and overexpression of truncated apoptotic isoform of Mcl-1 [61]. Stroma-derived factor 1a and CXCR4, as its cognate receptor, involve in the migration of hematopoietic cells to the bone marrow (125, 126 as 1). Binding of this ligand to the receptor plays an important role in resistance of leukemic cells to apoptosis after chemotherapy [62]. Therefore, AMD3100, as an inhibitor of CXCR4, prevents Akt phosphorylation and induction of PARP cleavage totally increasing the sensitivity of cancer cells to chemotherapy in leukemia [63]. As mobilization of hematopoietic stem cells depends on CXCR4, inhibition of CXCR4 makes multiple myeloma cells vulnerable to chemotherapy by disintegrating adhesion of myeloma cells to the stromal cells in the bone marrow [64]. In a PI3K/Akt manner, PD-L1 regulates stemness markers including OCT-4, NANOG, and BMI1 in breast cancer [65].

#### JAK/STAT pathway

Expression of different cytokines and growth factors to control proliferation, growth, and immune functions are regulated by JAK/STAT pathway. It is also involved in hematopoiesis, neurogenesis, and maintenance of self-renewal in embryonic stem cell. For example, STAT3 induces angiogenesis, immunosuppression, and tumor invasion, which all substantiate in progression of gliomagenesis [12]. IL-6/JAK2/STAT3 pathway are more active in breast CSCs compared with other cancer cells, and thereby, number of CSCs as well as growth of xenograft are decreased following JAK2 inhibition [66]. JAK/STAT signaling was active in CSCs of acute myeloid leukemia, and JAK2 inhibition hampers the growth of leukemic stem cells [67]. Self-renewal and tumorigenicity of glioblastoma stem-like cells were activated by FOXM1-PDGFA-STAT3 signaling [68].

#### NF-kB pathway

NF-kB is implicated in the regulation of innate and adaptive immunity. It is a fundamental mediator in inflammatory responses. NF-Kb regulates cell survival, activation, and differentiation of cells. Activation of NF-kB upregulates the expression of Interleukin 3 (IL-3) and granulocyte–macrophage colony-stimulating factor (GM-CSF), which in turn provokes the proliferation and survival of stem cells in leukemia [12]. In breast and lung cancer cells, NF-kB signaling activates LIN28/TCF4 interaction continued by promoting stemness and metastasis [69]. Breast cancer cells and CSCs highly express IL-8 after chemotherapy leading to the formation of an inflammatory loop between NF-kB and STAT3 signaling pathways [70]. Antagonizing Toll-like receptor-7 lowers the growth rate of CSCs in hepatocellular carcinoma through TLR7-IKK-NF-kB-IL6 signaling pathway [71].

#### Hippo-YAP/TAZ signaling

During normal organ development, this pathway controls cell fate and differentiation of progenitor cells [72]. Dedifferentiation and expansion of stem or progenitor cells are among the other functions of Hippo-YAP pathway [73]. Activation of YAP/TAZ signaling leads to the dedifferentiation of cancer cells to gain CSCs characteristics like self-renewal and chemoresistance [72]. The impact of YAP on the promoters of mammary stem cells results in the induction of breast CSCs [74]. Direct upregulation of SOX9 is performed by YAP that is known as a strong inducer of CSC properties [75]. Also, SOX2 activates YAP which maintain CSCs in osteosarcoma and glioblastoma [76]. Altogether, huge body of evidence confirm that YAP/TAZ significantly involve in the maintenance and progression of CSCs.

### Microenvironment

The microenvironment, in which division and differentiation of stem cells happen, controls stem cell functions through intercellular communication between stem cells and differentiated peers. Also, interactions of the cell with extracellular matrix, paracrine communication, hormone signaling, the effects of growth factors and mediators, and physicochemical characteristics of the microenvironment affect its components [29, 77]. These delicately acting signals protect and regulate normal stem cells. This is the case for CSCs as well. CSCs are exposed to a variety of growth factors and cytokines released by different types of stromal cells [78]. Plasticity of CSCs represents in reversible phenotypic changes like induction of expression of some markers upon microenvironment-related stimuli [79]. CSCs are also capable to recruit components of tumor microenvironment [80].

The microenvironment promotes CSCs survival in two ways: activation of specific molecular signaling pathways, and formation of a physical barrier to prevent penetration of therapeutic agents [8]. The cells present in the nearby microenvironment of CSCs instigate some signaling pathways like Notch and Wnt leading to metastasis, evasion from anoikis, and alteration of divisional property [9]. Other than helping CSCs to expedite their divisional dynamics by providing inherent properties of self-renewal and differentiation capabilities, microenvironment maintains CSCs in a primitive development state [2].

It is concluded that microenvironment is an influential factor that establishes a balance between self-renewal and differentiation of CSCs, possibly directing them toward proliferation, invasion, and metastasis [81]. Endothelial cells, cancer-associated fibroblasts, mesenchymal stromal/stem cells, inflammatory cells, and extracellular matrix constitute the tumor microenvironment. This pool of cells and mediators aids CSCs to grow and proliferate [79].

#### Cancer-associated fibroblasts

Fibroblast is the most prevalent component of tumor microenvironment especially in certain cancers like breast tumors. These cells are known as cancer-associated fibroblasts (CAFs) Coculture of breast cancer cells with CAFs increases resistance to tamoxifen up to 4.4 folds [8]. CAFs secrete a variety of growth factors, cytokines, and chemokines. Hepatocyte growth factor, as one of the secreting elements, activates MET receptor protecting CSCs against apoptosis following cetuximab therapy in colorectal cancer [82]. TGF-β is the most important mediator secreted by CAFs that promotes EMT and drug resistance [8]. It was reported that following paclitaxel treatment in breast cancer, TGF-β signaling and IL-8 expression are augmented, and thereby, population of CSC is enriched leading to tumor recurrence [83].

CAFs increase secretion of some cytokines and chemokines, support self-renewal and invasiveness of CSCs, and ultimately provide chemoresistance [84]. Exosomes that secreted from CAFs help resistance to 5-fluorouracil in colon cancer [85]. Some other mediators like Neuregulin 1 (NRG1) are secreted by CAFs, which provoke stemness and activate NF-KB signaling. Type I collagen is also secreted by CAFs, and decreases drug influx [8]. On the other hand, CSCs have the ability to differentiate into CAFs-like cells leading to tumor maintenance and survival [86]. Chemicals that specifically target CAFs show their therapeutic benefits in the treatment of breast cancer [87].

#### Immune cells

CSCs directly attenuate immune surveillance facilitating tumor growth [88]. There are many reports regarding the contribution of inflammatory cells like monocytes and macrophages to the tumor microenvironment [89]. Macrophages, dendritic cells, myeloid-derived suppressor cells, and other components of the innate immunity regulate CSCs and tumor growth [90]. Macrophages in the tumor microenvironment are called tumor-associated macrophages (TAMs) or M2 macrophages. CSCs release proinflammatory cytokines and chemokines in order to recruit macrophages [8]. Once reaching to the tumor niche, macrophages transform into TAMs. Some cytokines like CCL2 promote infiltration of TAMs into the tumors, in primary or even metastatic regions. Interaction of TAMs with tumor cells is mediated through a variety of factors such as TGF-β and TNF-α that stimulate EMT process [8]. Cancer cells of pancreatic adenocarcinoma secrete colony-stimulating factor 1. This is followed by attraction and stimulation of its receptor on TAMs [91]. TAMs increase the expression of cytidine deaminase that catabolizes gemcitabine. TAMs also activate certain signaling pathways in CSCs such as STAT3 and Hedgehog via secretion of some cytokines like milk fat globule epidermal growth factor 8 and Interleukin-6 (IL-6) totally providing chemoresistance. Activation of STAT3 by TAMs inhibits antitumor responses raised by CD8^+^ T lymphocytes, and provokes characteristics of CSC in pancreatic tumor cells [92]. Thus, TAMs are potent targets for downregulation of tumor-initiating cells. Another player among immune components is dendritic cells that makes a bridge between innate immunity and adaptive immunity. These cells are capable of inducing chemoresistance and tumorigenesis [12].

#### Mesenchymal stem cells

One type of adult stem cells is mesenchymal stem cells (MSCs). Under normal conditions, MSCs act as immunomodulators, and differentiate into specialized cells supporting hemostasis. In the tumor microenvironment, MSCs activate NF-KB signaling that induces CSC phonotype and chemoresistance [29]. It was shown that abnormal DNA hypermethylation of the two tumor suppressor genes (HIC1 and RassF1A) change MSCs to CSCs causing resistance to cisplatin [93]. Breast CSCs secrete IL-6 that recruits MSCs, which in turn leads to CXCL7 release. This cytokine accelerates tumor growth and chemoresistance in animal models. Also, it was shown that IL-6 activates STAT-3 signaling that enriches CSC population, and results in trastuzumab resistance in breast cancer. CSCs are able to differentiate into endothelial cells, and endothelial cells differentiate into MSCs suggesting that CSCs could generate MSCs [8]. Based on higher expression of integrin-linked kinase in mesenchymal cells compared with epithelial cells, the former possess elevated expression of genes involved in metastasis and invasion, and accordingly, they become resistant to some chemotherapy drugs like erlotinib, gefitinib, and cetuximab [94]. Trastuzimab resistance was gained by PTEN downregulation and c-Src activation following coculturing of breast cancer cells and MSCs [95].

#### Extracellular matrix

Extracellular matrix (ECM) is constituted from a population of molecules that mostly produced by fibroblasts. Cancer cells attach to the ECM in order to form a tumor. The high stiffness of ECM in solid tumors is like a physical barrier that protect CSCs to be reached by therapeutic drugs. Furthermore, proteins of the ECM interact with membrane proteins of CSCs. This interaction activates signaling pathways involved in stemness, proliferation, and eventually chemoresistance [8].

#### Endothelial cells and oxygen content

Oxygen and nutrients are delivered to the tumor microenvironment by blood vessels. It was shown that this vasculature plays an important role in maintaining the properties of CSCs like self-renewal. Endothelial cells of the vascular system release several growth factors such as EGF, which induces EMT process and CSC characteristics in many tumors [8]. Irregular shape of blood vessels in the tumor microenvironment hampers drug delivery to the CSCs [96]. CSCs themselves show a complex interaction with the endothelial cells. CSCs recruit, and also able to directly differentiate into endothelial cells. It was shown that hypoxia and glucose shortage are inducers of CSCs differentiation into endothelial cells. So, maintenance and development of self-renewal in CSCs are influenced by the hypoxic condition [8]. Interestingly, CSCs have a tendency to be in a close proximity of the hypoxic regions within the tumors [97]. In fact, certain features of CSCs are expressed in the hypoxic conditions [98].

Hypoxia provokes drug resistance via activation of stem-related pathways and promotion of quiescence. Activation of hypoxia-inducible factor (HIF)-1α leads to the expression of many effectors involving in EMT process like Wnt, Hedgehog, and Notch pathways besides upregulation of some stemness markers [99]. Hypoxia contributes to CSCs enrichment via hyperactivation of VEGF, IL-6, and stemness-related genes like NANOG, OCT4, and EZH2, as shown in pancreatic tumor cells [100]. Increasing the expression of insulin-like growth factor 1 due to hypoxia through HIF1α and activation of IGF1 receptor induce resistance to gefitinib in lung CSCs [101].

Cellular responses to hypoxia are mainly regulated by HIF-1α, which is degraded at high oxygen levels. In low oxygen tension, it becomes activated, moves to the nucleus, undergoes dimerization with HIF-1β, and eventually, stimulates the expression of response elements in 60 genes that are important in angiogenesis, oxygen delivery, and activation of survival pathways. HIF-1α decreases reactive oxygen species (ROS) as well. This function results in drug resistance and induction of quiescence in CSCs [8]. Cellular growth is retarded and quiescence is induced in the tumor cells in the conditions of low oxygen and nutrients deprivation [80].

### ATP-binding cassette transporters

Protecting normal stem cells is a vital task because these cells are biologically necessary to support the pool of cells in different tissues. Therefore, stem cells are equipped with several mechanisms to avoid apoptosis or senescence. Meanwhile, CSCs use the same mechanisms to repel anti-cancer therapeutic drugs like cisplatin, paclitaxel, docetaxel, and cetuximab [102]. There are protein in the cell membrane called ATP-binding cassette (ABC transporters) that efficiently translocate molecules across the membrane. Rapid transportation of chemotherapeutic agents from inside to the extracellular space causes multidrug resistance in CSCs [103]. Some believe that this may be the most powerful resistance strategy that is used by CSCs [104].A great number of these proteins are expressed on the cell surface of CSCs. Of the important ones are ABCB1, ABCG2, ABCB5, and ABCC1. Staining a population of tumor cells with Hoechst 33342 dye and Rhodamine 123 dye helps to identify overexpressed ABC transporters, and consequently, determine the location of CSCs within a tumor [8]. Notably, stemness markers like c-Myc are involved in CSCs chemoresistance by increasing the expression of ABC transporters. CD44, another stemness marker, activates chemoresistance through anti-apoptotic proteins and ABC transporters [2].

ABCG2/ABCB1 transporters are highly expressed in hematopoietic stem cells [105]. Coexpression of ABCG2 and CD133 is a hint for identification of tumor-initiating cells in melanoma cancer. Progression of melanoma is clinically correlated with ABCB5 expression. Rather than other stem cell markers, simultaneous expression of ABCB1, ABCB5, and ABCC2 were seen in a subpopulation of melanoma cells [106]. Side population cells in glioma that efflux Hoechst 33342 dye and express ABCG2 transporter through PTEN/PI3K/Akt signaling are strongly tumorigenic, and show resistance against temozolomide [60].

### Dormancy

In CSCs, cell death mechanisms are controlled via modulation of cell cycle. This strategy, which also contributes to EMT, creates resistance. While conventional cancer therapy mainly targets cancer cells relying on their rapid proliferation, slow cycling-cancer cells are not affected [12]. CSCs maintain themselves in a quiescent state resulting in an inherent resistance to such treatments [107], and are found in melanoma, glioblastoma, squamous cell carcinoma, and bladder cancer [108].

Such cells promote long-term tumor growth, and generate a cell population with highly proliferative potential eventually leads to cancer relapse [109]. Furthermore, the imposed injuries caused by chemotherapy drugs recruit quiescent cells during the interval between treatment cycles resulting in repopulation of tumor cells [110].

Genetically distinct subclones produced by heterogeneous population within a tumor have diverse functions [111]. Moreover, previously dormant or slow-proliferating cancer cells are activated after chemotherapy. Totally these situations reduce the treatment efficacy and increase the risk of cancer relapse [111]. Cancer relapse several years after initial treatment reveals that CSCs could survive through the dormant state during treatment course and reactivate afterward [112].

### Ferroptosis

Ferroptosis is a form of non-apoptotic cell death. It differs from apoptosis, necrosis, and autophagy as ferroptosis is an important type of cell death in cancer cells. It occurs as a result of imbalance between lipid hydroperoxides (possibly due to overproduction of lipid ROS) and specific detoxification enzymes. Lipid metabolism, ROS production, and iron addiction are different between cancer cells and normal peers. CSCs, similar to other cells, metabolize lipid, though in a higher degree, in order to provide enough energy for their different functions. In CSCs, cytoplasmic organelles called lipid droplets are generated for lipid storage. Lipids are protected in these organelles from peroxidation, and this causes resistance against ferroptosis. Lipid desaturation is the other protective mechanism against lipid peroxidation in CSCs. Conversion of saturated fatty acids to mono-unsaturated fatty acids (MUFAs) prevents ferroptosis because MUFAs reduce ROS and poly-unsaturated fatty acids (PUFA)-containing phospholipids. Lipid desaturation is regulated by stearoyl-CoA desaturase 1. This enzyme along with lipid droplets are implicated in stemness of CSCs [2].

CSCs are unique with respect to iron metabolism due to their unusual expression of some proteins involved in iron import, export, and storage [2]. For instance, ferritin as the iron storage protein, is upregulated in CSCs, and consequently, CSCs have higher iron level. This intracellular storage of iron augments stem-related characteristics like proliferation and maintenance [2]. It was shown that low level of iron is associated with downregulation of EMT markers like E-cadherin in breast cancer cells [113].

### Autophagy

Autophagy is a fundamental determinant for CSCs aggressiveness. In fact, autophagy is a bidirectional road that promotes survival or death by either sustaining the cell viability or activating a phagosome-lysosome-dependent feature. Cellular context determines direction of the cell toward each pathway. Autophagy is able to alter the microenvironment in favor of CSCs by making an imbalance between CSCs and normal cells. Autophagy also instigates ferroptosis to induce cell death via degradation of ferritin [2]. In the case of hypoxia and nutrient deprivation in the microenvironment, autophagy is increased [114].

### Reactive oxygen species

One of the main reasons of chemoresistance in CSCs is the low content of ROS [115]. Scavenging system in CSCs is strong enough to keep the intracellular ROS at a level similar to that of normal stem cells [116]. Breast CSCs demonstrate enhanced expression of scavenging genes like superoxide dismutase, glutathione peroxidase, and catalase [116–118]. Activation of oncogenic transcription factors like c-Myc increases ROS levels followed by NRF2 activation. NRF2 is an important transcription factor that upregulates genes involved in detoxification and antioxidant activity such as NADPH quinone oxidoreductase (NQO-1), glutathione (GSH), and glutathione peroxidase (GPX) [2]. Indeed, NRF2 activates the expression of efflux transporters and anti-apoptotic proteins like BCL-2 [119]. Also, it was shown that NRF2 contributes considerably to iron homeostasis [120]. In this regard, NRF2 could be considered as a promising target in CSCs therapy [2].

High NRF2 level in CSCs is related to the high expression of some markers like CD44 and ALDH [121]. Enhanced expression of CD44 translates into higher GSH synthesis, and thereby, stronger shield against ROS [122]. Some cancer cells like those in oral cavity have high contents of antioxidant enzymes such as SOD2 and peroxiredoxin 3. This leads to low levels of ROS representing in resistance to cisplatin [123]. Another protecting mechanism of CSCs is oxidation of intracellular aldehyde by ALDH in order to prevent ROS-induced cell injury [124]. In normal condition, ALDH has diverse functions such as acetaldehyde oxidation, cellular detoxification, and regulation of stem cell tasks [117]. ALDH activity is considered as a selective marker for identification of CSCs in many types of tumors [125]. The main isoform of ALDH is ALDH1. This isoform has a detoxifying function by concurrent reduction of ROS and generation of antioxidant compounds like NADP. ALDH1 also keeps cells safe against alkylating agents like paclitaxel [126]. It was shown that ALDH1A1 and ALDH3A1 are overexpressed in the cancer cells [117]. Also, ALDH1A1 augments the activation of DNA repair mechanism in such cells [127]. Elevated expression of ALDH1 is a predictor of poor response and prognosis in esophageal cancer [128]. CSCs that express ALDH1A1 are significantly resistant to gefitinib, cisplatin, etoposide, and fluorouracil in the lung tissues [129].

Activation of DNA damage responses increases the number of CSCs to about 2–4 folds [8]. In glioma, activation of DNA damage checkpoints is more effective in CD133^+^ cells after radiation compared with CD133^−^ peers [15]. DNA repair mechanisms are enhanced in glioblastoma stem cells compared with progenitor cells [130]. So, glioblastoma stem cells profoundly become sensitive by inhibition of PARP and ATR [131]. Overexpression of polymerase η gives CSCs resistance to cisplatin in ovarian cancer cells, and sensitivity of CSCs to cisplatin is increased by activating mir-93, which regulates the expression of polymerase η [132].

However, response to DNA damage has opposing consequences. Normal stem cells use this strategy in order to provide optimal functions in healthy tissues while leads to CSCs survival and resistance. High levels of replication stress are tolerated by CSCs via this mechanism. Even, CSCs resist against chemical therapeutics that are specifically designed to damage DNA [131].

### Other mechanisms

There are other mechanisms that give CSCs resilience [2]. Epigenetic changes including elevation of the euchromatin mark H3-lys4 trimethylation, reduction of the heterochromatin mark H3-lys9 dimethylation, and increasing transcriptional mark H3-lys36 trimethylation are happened during TGF-β mediated EMT. These alterations, particularly the first one, implicate in chemoresistance [133]. EZH2, a PcG member and a subunit of polycomb repressor complex 2 that provokes gene silencing by histone trimethylation, participates in pancreatic cancer chemoresistance via turning off the p27 tumor suppressor gene [134]. In chronic myelogenous leukemia, quiescent stem cells that are resistant to imatinib mesylate are effectively eliminated after treatment with inhibitors of histone deacetylase [135].

Binding of NANOG to the promoter region decreases K14 and K27 histone H3 acetylation. This event activates histone deacetylase 1, and represses cell cycle inhibitors of CDKN2D and CDKN1B inducing stem-like features. Also, silencing of E3 ubiquitin-ligase TRIM17 and NOXA upregulate MCL-1 facilitating immune resistance and chemoresistance [136]. Complete hypermethylation of the hMLH1 promoter leads to loss of DNA mismatch repair gene [137]. This arrests cell death and cell cycle after chemotherapy-induced DNA damage and consequently, leads to low rate of survival in ovarian and breast cancer patients [138].

MicroRNAs are non-coding RNAs that have roles in chemoresistance, EMT activation, and acquisition of CSCs characteristics [139]. Repression of PI3K signaling by mir-126 increases self-renewal and decreases differentiation ability in leukemic stem cells through keeping them in a quiescent state and restricting their entry to the cell cycle [140]. By targeting PTEN, mir-10b regulates expression of stem cell markers in breast cancer lines [141].

CSCs produce different proinflammatory signals like Interferon (IFN) regulatory factor-5, a transcription factor that specifically induces chemoresistance via induction of macrophage colony-stimulating factor (M-CSF) [142]. Significant expression of IFN regulatory factor-5 by CSCs is assumed to be necessary for tumorigenic activities in myeloid cells [142]. In some cases, CSCs use inflammatory mediators to resist against therapeutic drugs. For instance, breast CSCs use IL-6 inflammatory loop to resist against trastuzumab [143]. Telomere shortening causes chromosome instability, cell fusion, and senescence. Telomerase, an enzyme that adds repeats to the telomeres end, is active in tumor cells, and give them self-renewal. Telomerase activity was found in nearly 90% of breast carcinoma cells while it was completely absent in breast normal cells [8].

## Therapeutic strategies

**Fig. 2:**
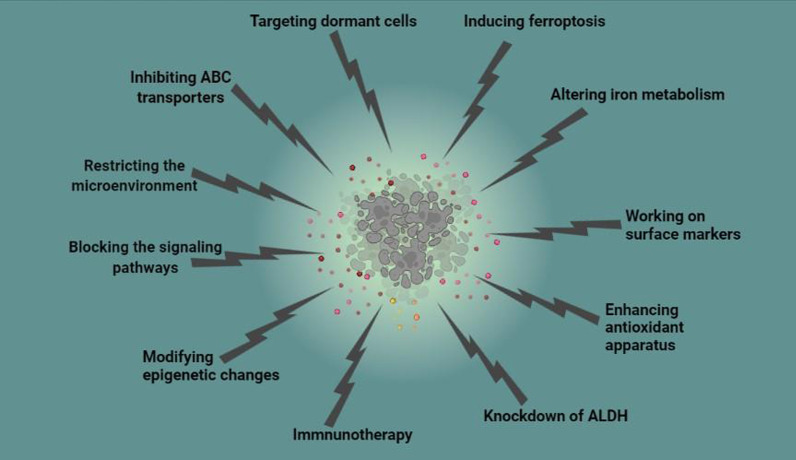
Specific therapeutic strategies for elimination of cancer stem cells

Conventional chemotherapy eliminates bulk of proliferating cancer cells, but CSCs survive and move to a higher level of invasiveness and chemoresistance. On the other hand, chemotherapy provokes heterogeneity in both normal and cancer cells that consequently attenuates treatment efficiency. Higher expression of CSC biomarkers in the tumor is associated with poor prognosis [2]. Different therapeutic approaches have been designed to sensitize Achilles’ hill of the tumor, CSCs. However, these advancements though promising are not desirable possibly because they are not fine-tuned for eradication of CSCs [2]. Hence, the way toward finding effective cancer treatment is still in its infancy, and needs more developments for future treatment plans (Table 1). Here, we describe some of the important and undergoing strategies that specifically target mechanisms used by CSCs for resistance (Fig. 2).

**Table 1:** Interventional clinical trials targeting cancer stem cells since 2020

Identifier	Condition or disease	Intervention/treatment	Number of participants
NCT02423811	Esophageal squamous cell carcinoma	Fursultiamine (a dietary Supplement) in addition to Concurrent chemoradiotherapy	20
NCT03949283	Recurrent Ovarian Carcinoma; Platinum-resistant Ovarian Cancer	Standard Chemotherapy Versus Cancer Stem Cell Assay Directed Chemotherapy	220
NCT03632798	Recurrent Ovarian Cancer	Avastin Plus Chemotherapy vs. Avastin Plus Chemotherapy Chosen by Cancer Stem Cell Chemosensitivity Testing	300
NCT03632135	Recurrent Glioblastoma	Standard Chemotherapy Versus Chemotherapy Chosen by Cancer Stem Cell Chemosensitivity Testing	300
NCT02654964	Glioblastoma Multiforme	Combination Drug Therapy Based on Personalized Cancer Stem Cell High-Throughput Drug Screening	10
NCT02089919	Metastatic Adenocarcinoma of the Liver	Cancer Stem Cell Vaccine	40
NCT02074046	Metastatic Adenocarcinoma of the Pancreas	Cancer Stem Cell Vaccine	40
NCT02115958	Lung Neoplasms (Metastatic of the Nasopharynx)	Cancer Stem Cell Vaccine	40
NCT02084823	Metastatic Adenocarcinoma of the Lung	Cancer Stem Cell Vaccine	40
NCT02178670	Metastatic Adenocarcinoma of the Ovarian	Cancer Stem Cell Vaccine	40
NCT02176746	Metastatic Adenocarcinoma of the Colorectal	Cancer Stem Cell Vaccine	40
NCT03548571	Glioblastoma	Dendritic Cell Immunotherapy	60
NCT01190345	Breast Cancer	Pre-operative Bevacizumab in Combination With Chemotherapy	75
NCT01579812	Ovarian, Fallopian Tube, and Primary Peritoneal Cancer	Metformin	90
NCT01440127	Colon Cancer	Metformin	9
NCT02859415	Esophageal Neoplasms, Lung Neoplasms, Mesothelioma, Thymus Neoplasms, Germ Cell and Embryonal Neoplasms	Continuous 24 h Intravenous Infusion of Mithramycin	60
NCT01118975	Breast Cancer Neoplasm Metastasis	Vorinostat and Lapatinib	12
NCT01624090	Lung Cancer, Esophageal Cancer, Mesothelioma, Gastrointestinal Neoplasms, Breast Cancer	Mithramycin	16
NCT01195415	Recurrent Pancreatic Carcinoma, Stage IV Pancreatic Cancer	Gemcitabine Hydrochloride and Vismodegib	25
NCT01255800	Recurrent Head and Neck Cancer	Cetuximab and the Hedgehog Inhibitor IPI-926	9
NCT01868503	Locally Advanced or Locally Recurrent Breast Cancer	Concurrent Lapatinib and Radiotherapy	7
NCT01334047	Recurrent Epithelial Ovarian Cancer	Autologous Dendritic Cells Loaded With Amplified Ovarian Cancer Stem Cell mRNA, hTERT and Survivin (Vaccine therapy)	5
NCT02370238	Metastatic Breast Cancer	Paclitaxel in Combination With Reparixin Compared to Paclitaxel Alone	156
NCT01861054	Breast Cancer	Reparixin	
	20		
NCT02001974	Metastatic Breast Cancer	Paclitaxel + Reparixin	33
NCT00645333	Metastatic Breast Cancer	MK-0752, Docetaxel, and Pegfilgrastim	30
NCT02775695	Resectable Pancreatic Cancer	Doxycycline	12
NCT02010606	Recurrent Glioblastoma	Vaccination With Autologous Dendritic Cells Pulsed With Lysate Derived From an Allogeneic Glioblastoma Stem-like Cell Line	39

### Blocking the signaling pathways

Blocking vital pathways of CSCs like Notch, Wnt, and Hedgehog that are necessary to their self-renewal would be a favorable approach [35]. Some small molecules such as evodiamine, IGC-001, and acridine derivatives are developed to deactivate CSCs through Wnt inhibition [144]. Salinomycine and its derivative, ironomycin, are inhibitors of Wnt/β-catenin pathway that also interfere with ABC drug transporters [8]. It was shown that Notch and Hedgehog signaling pathways are inhibited by Honokiol and cyclopamine, respectively [8]. γ-secretase inhibitors are among the most important class of Notch blockers [117]. Vismodegib is an inhibitor of Hedgehog pathway that was approved by FDA [8]. JAK/STAT pathway is downregulated by molecules such as EC-70124 and napabucasin [144]. One of the therapeutic strategies against CSCs could be delivered through targeting EMT pathway [145]. Three general target groups have been designed in this regard. These include regulators of EMT extracellular inducers and extracellular matrix components, certain transcription factors that promote EMT transcriptome and downstream effectors, and regulators of EMT-related transcription factors and epigenetic effectors [146].

### Restricting the microenvironment

Surprisingly, deactivation of CSCs solely does not guarantee tumor eradication. Tumor microenvironment, which harbors different cells and multiple factors, enhances survival, plasticity, and drug resistance of CSCs. Therefore, targeting tumor microenvironment increases CSCs sensitivity [80]. To substantiate, differentiation of non-CSCs into CSCs is done through reprogramming processes like EMT [8]. Components of tumor microenvironment like CAFs or TAMs release EMT inducers [8]. ECM and related proteins are valuable targets in the era of CSCs eradication. Destruction of hyaluronic acid and subsequent reduction in stroma reduce interstitial pressure while increasing the expansion of the vasculature; these totally augment the accessibility of chemotherapy drugs to CSCs [147]. CSCs also use their microenvironment to escape from immune surveillance. In this regard, components of microenvironment like CAFs are suitable targets in order to make CSCs available for immune system [148]. Also, innate immune cells release protumorigenic factors during chronic inflammation. It is important especially in those cancers (e.g. colon cancer) that inflammation plays a significant role. Hence, anti-inflammatory anti-cyclooxygenase-2 considerably decreases the risk of colon cancer [149].

### Inhibiting ABC transporters

Making ABC transporters dysfunctional in tandem with restoration of apoptosis pathways sensitizes CSCs to chemotherapy [150]. Metformin showed promising results for attenuating resistance via inhibition of an ABC transporter [8].

### Targeting dormant cells

Induction of activation in dormant CSCs to activate and commence the cell cycling increases their vulnerability to certain therapies. Meanwhile, efforts are undergoing to specifically identify and target dormant CSCs [151].

### Inducing ferroptosis

Activation of ferroptosis offers a safe treatment because cancer cells are more susceptible to ferroptotic death in comparison with normal cells. Ironomycin, ebselen, and other pyrazole and benzylisothiourea-containing agents as well as artemisinin derivatives are among the many ferroptosis inducing drugs that specifically target CSCs. Combinatorial therapy of ferroptosis inducing agents like erastin with chemotherapeutic agents like cisplatin, doxorubicin, and temozolomide increases treatment efficiency synergistically. However, different types of cancer cells show varying degrees of vulnerability to this type of death. Some cancer types like that of the breast, colon, and lung are more resistant to ferroptosis than some others like renal cell carcinoma and diffuse large B cell lymphomas. This variety may be partly explained by different degrees of aggressiveness of the tumors [2].

### Enhancing antioxidant repertoire and altering iron metabolism

One less studied fact about CSCs is overexpression of antioxidant and detoxifying genes as well as aberrant metabolism of iron and lipid [152]. Some researchers suggested that targeting iron metabolism in CSCs is a good approach for cancer therapy. Salinomycin sequesters iron in lysosomes, degrades ferritin, and promotes the Fenton reaction totally produces lipid peroxides leading to ferroptosis [153].

### Working on surface markers

Surface markers of CSCs are potential targets [154]. For instance, CD44 and CD133, surface biomarkers of CSCs, are targeted by H90 and oxytetracycline [155]. However, lack of specific markers in CSCs hinders the efforts to reach favorable outcomes [8]. Unfortunately, surface markers of CSCs in different tumor types are not similar [154]. Another challenging issue is that these markers are also expressed by normal cells [154].

### Knockdown of ALDH

Targeting ALDH is an alternative choiceto eliminate CSCs [8]. CSCs become vulnerable to cyclophosphamide after knockdown of two isoforms of ALDH [156]. High intracellular level of retinoic acid decreases the expression of ALDH. Therefore, all-trans retinoic acid is a potent therapeutic agent in acute promyelocytic leukemia. Combination of all-trans retinoic acid with retinoids suppress ALDH activation in a synergistic manner [117].

### Immnunotherapy

Immunotherapy is considered as a breakthrough in cancer therapy that specifically targets CSCs within a tumor entity. In this type of treatment, several components of the immune system including natural killer (NK) cells and *γδ*T cells of the innate immunity, antibodies of acquired humoral immunity, CSC-based dendritic cells, and CSC-primed cytotoxic T lymphocytes (CTLs) of acquired cellular immunity recognize and kill CSCs-associated molecules. Finding specific targets such as appropriate antigens like markers of CSCs, antigens involved in the interactions of CSC with microenvironment, cytokines, and immune checkpoint, and genetic alterations in CSCs are of eminent importance in immunotherapy [157].

### Modifying epigenetic changes

Epigenetic changes like histone acetylation and DNA methylation affect the regulation of drug resistance in CSCs [9]. CSCs are associated with aberrant expression of histone deacetylase (HDAC) [158]. Inhibition of HDAC and targeting histones per se are assumed as the therapeutic strategies against CSCs [84]. HDAC inhibitors like vorinostat suppress CSCs both in in vitro and in vivo experiments of different tumor cells. DNA methylation inhibits tumor suppressor genes promoting chemoresistance. For instance, hypermethylation of promoter region of insulin-like growth factor binding protein-3 downregulates its expression, and consequently, modifies cell growth, transformation, and survival toward enhancing chemoresistance [9].

### Other strategies

While a growing body of evidence revealed that miRNA and other long noncoding RNAs substantially contribute to the regulation of vital CSCs characteristics like self-renewal, asymmetric cell division, tumor initiation, drug resistance, and tumor relapse [159], targeting such miRNAs is considered in CSCs-based therapy. Also, CSCs possess a strict dependence on the biogenesis of mitochondrion, which is inhibited by doxycycline. In this way, doxycycline sensitizes CSCs to paclitaxel as well. Doxycycline reduces metastasis in breast cancer along with attenuating the tumor burden in pancreas cancer [8]. Of other strategies is the development of some drugs like atovaquone and artesunate that inhibit oxygen consumption and induce mitochondrial dysfunction in CSCs [160].

### Limitations

Although these novel strategies seem efficient especially in combination with conventional therapeutics, challenges still hamper the desirable goal. Some of the novel drugs may be toxic due to lack of specificity as there are similarities in markers and pathways between CSCs and normal stem cells [33]. Another important issue is the heterogeneity within a tumor. This causes different cells to express different markers with different dysregulated pathways [148]. Hence, designing a single agent that efficiently destroys CSCs despite all of these diversities is a laborious task if possible at all. Therefore, seeking the unique characteristic of CSCs, which are absent in normal stem cells as well as other vital contributors, is the gate of success toward elimination of cancer cells.

## Conclusion

Major therapeutic advancements have been reached in the battle against cancer. However, certain properties of the tumors that are mainly derived from CSCs challenge optimal treatment. Targeting this population of cancer cells is of utmost essence. To reach this goal, obtaining an in-depth understanding of the mechanisms that make CSCs alive despite anticancer therapy is very critical. Implementation of the strategies that discriminate between CSCs and other normal peers advance this road forward significantly.

## Data Availability

Not applicable.

## Abbreviations

CSCs: Cancer stem cells
SCs: Stem cells
ALDH1: Aldehyde dehydrogenase 1
EMT: Epithelial-mesenchymal-transition
JAK-STAT: Janus kinase-signal transducer and activator of transcription
NRF2: Nuclear factor erythroid 2-related factor 2
PTEN: Phosphatase and tensin homolog
CAFs: Cancer-associated fibroblasts
TAMs: Tumor-associated macrophages
MSCs: Mesenchymal stem cells
ECM: Extracellular matrix
HIF: Hypoxia-inducible factor
ROS: Reactive oxygen species
ABC transporters: ATP-binding cassette
MUFAs: Mono-unsaturated fatty acids
PUFA: Poly-unsaturated fatty acids
NQO-1: NADPH quinone oxidoreductase
GSH: Glutathione
GPX: Glutathione peroxidase
NK: Natural killer
CTLs: Cytotoxic T lymphocytes
HDAC: Histone deacetylase
YAP/TAZ: Yes-associated protein 1/Transcriptional coactivator with PDZ-binding motif
c-Myc: Cellular Myc
SOX2: SRY-Box Transcription Factor 2
OCT4: Octamer-binding transcription factor 4
TGFβ: Transforming growth factor beta
PI3K/Akt: Phosphatidylinositol 3-kinase/protein kinase B
EGFR: Epidermal growth factor receptor
NF-kB: Nuclear factor kappa light chain enhancer of activated B cells
IL-3: Interleukin 3
GM-CSF: Granulocyte-macrophage colony-stimulating factor
NRG1: Neuregulin 1
IL-6: Interleukin-6
IFN: Interferon
M-CSF.: Macrophage colony-stimulating factor
